# The effects of ankle dorsiflexor fatigue on lower limb biomechanics during badminton forward forehand and backhand lunge

**DOI:** 10.3389/fbioe.2023.1013100

**Published:** 2023-01-30

**Authors:** Jianhua Tong, Zhenghui Lu, Xuanzhen Cen, Chaoyi Chen, Ukadike Chris Ugbolue, Yaodong Gu

**Affiliations:** ^1^ Faculty of Sports Science, Ningbo University, Ningbo, China; ^2^ Doctoral School on Safety and Security Sciences, Obuda University, Budapest, Hungary; ^3^ School of Health and Life Science, University of the West of Scotland, Scotland, United Kingdom; ^4^ Research Academy of Medicine Combining Sports, Hwa Mei Hospital, University of Chinese Academy of Sciences, Ningbo, China

**Keywords:** badminton, muscle fatigue, lunge, biomechanics, lower limb

## Abstract

**Background:** Local muscle fatigue may have an adverse effect on the biomechanics of the lunge movement and athletic performance. This study analyzed the biomechanical indicators of the forward lunge in badminton players before and after fatigue of the ankle dorsiflexors.

**Methods:** Using the isometric muscular strength testing system, 15 badminton players underwent an ankle dorsiflexor fatigue test. Before and after the fatigue experiment, five lunges were done in both the forehand forward (FH) and backhand forward (BH) directions, five in each direction. A Vicon motion capture system and an AMTI force measuring station were used to record lower limb kinematic and ground reaction force (GRF). Pre-fatigue and post-fatigue variability were determined using paired-samples t-tests, Wilcoxon signed rank test, and Statistical Non-parametric Mapping (SNPM).

**Result:** The results showed that after fatigue, the peak angle of ankle dorsiflexion was significantly reduced (*p* = 0.034), the range of motion (ROM) of the ankle sagittal plane (*p* = 0.000) and peak angle of ankle plantarflexion (*p* = 0.001) was significantly increased after forehand landing. After fatigue, ankle inversion was significantly increased after forehand and backhand landings (FH: *p* = 0.033; BH: *p* = 0.015). After fatigue, peak knee flexion angles increased significantly (FH: Max: *p* = 0.000, Min: *p* = 0.000; BH: Max: *p* = 0.017, Min: *p* = 0.037) during forehand and backhand landings and ROM in knee flexion and extension increased (*p* = 0.009) during forehand landings. Knee inversion range of motion was significantly increased after fatigue (*p* = 0.024) during forehand landings. Peak hip flexion angle (*p* = 0.000) and range of motion (*p* = 0.000) were significantly reduced in forehand landings after fatigue. The mean loading rate (*p* = 0.005) and the maximum loading rate (*p* = 0.001) increased significantly during backhand landings after fatigue. Post-fatigue, the center of pressure (COP) frontal offset increased significantly (FH: *p* = 0.000; BH: *p* = 0.000) in the forehand and backhand landings.

**Conclusion:** These results indicate that when the ankle dorsiflexors are fatigued, the performance of the forehand is significantly negatively affected, and the impact force of the backhand is greater.

## 1 Introduction

Badminton is one of the most popular sports in the world, and it is a non-contact racket sport. Participants in badminton need to perform running, jumping, stopping abruptly, and lunging (Shariff et al., 2009; Kuntze et al., 2010; Phomsoupha and Laffaye, 2015), with the lunge accounting for 15% or more of the total number of movements in a single game (Lam et al., 2020). Due to the rapid and violent impact, the lower limbs are subjected to a greater load than walk or run during the lunge heel landing phase of badminton, which may increase the risk of damage to the lower limbs (Cronin et al., 2003; Robinson and O’Donoghue, 2008; Lam W. K. et al., 2017; Dempster et al., 2021). The rate of injury per badminton player is 0.85 per year, and the proportion of lower limb injuries is approximately 58% (Boesen et al., 2011). The ankle and knee joints account for the majority of lower limb injuries among badminton players, whereas a well-executed lunge increases the deceleration of ground reaction forces (GRF) and the stability of the lower extremity landing position, thus reducing the possibility of ankle and knee injuries (Krøner et al., 1990; Valldecabres et al., 2020a).

During the lunge landing, the athlete’s lower limb joints are subjected to heavy loads and adapt to rapid changes in body posture, which exerts greater demands on their muscle strength, ability to absorb stress in the lower limbs, and lower limb joint stability than general body sports (e.g., running, walking) (Huang et al., 2014; Al-Nuaim and Safi, 2022; Xiang et al., 2022; Xu et al., 2022). In addition, fatigue has negative effects on athletic performance as well as the coordination and precision of motor postural control (Kellis and Liassou, 2009), which has a significant impact on the participation experience of athletes and is a major cause of injury.

Systemic fatigue has been shown to increase ankle inversion angle and knee stiffness during typical badminton lunge landings (Valldecabres et al., 2018; Herbaut and Delannoy, 2020), thereby increasing the risk of ankle sprains and knee loading in badminton players. It has been revealed that the angle of the ankle and knee joints during landing is also a significant determinant of joint stability (Bates et al., 1978). However, current fatigue protocols rarely link muscle fatigue to biomechanical changes in badminton (Sarshin et al., 2011; Valldecabres et al., 2020b; Herbaut and Delannoy, 2020). Local muscle fatigue may have effects exercise performance and loading, causing the stress distribution on the musculoskeletal structure to change (RADIN, 1986; Christina et al., 2001; Kellis and Liassou, 2009; Tiwari et al., 2021; Yahya et al., 2022). As a major player in ankle motion, the dorsiflexors use concentric contraction to increase dorsiflexion before landing in a lunge to provide adequate landing cushion range (Kim et al., 2017). With a greater range of motion (ROM) in the ankle dorsiflexion, the body can take less impact when landing and be more cushioned. During the landing phase of the lunge, the dorsiflexors alternate with the plantar flexors; although the plantar flexors are dominant, the dorsiflexors also have an irreplaceable role. It has been shown that when the knee is flexed beyond 90°, the plantar flexors’ eccentric contraction increases during lunge landing, but the dorsiflexors also increase their activity and contribute to ankle stability (Lees and Hurley, 1994; Kim et al., 2017; Lee and Loh, 2019). Furthermore, the effect of dorsiflexor fatigue on ankle motion during landing is currently unknown. In previous research on local muscle fatigue, it was found that dorsiflexor fatigue increased the magnitude of postural sway and impaired dynamic postural stability, thereby increasing the risk of ankle injury (Lundin et al., 1993).

Previous studies have been conducted on the impact of ankle dorsiflexor fatigue on the kinematics, kinetics, and stability of the lower extremities during running (Lundin et al., 1993; Flynn et al., 2004; Kellis and Liassou, 2009; Mattes et al., 2015). The effects of dorsiflexor fatigue are currently unclear on badminton lunge motions. In badminton singles, players frequently lunge forward to hit the shuttlecock, accounting for approximately 37% of all movements (Hu et al., 2015; Phomsoupha and Laffaye, 2015). On the forward lunge, previous studies have found that the lower limb joint loads and plantar pressures vary based on the lunge’s direction (Hong et al., 2014; Hu et al., 2015). Forward forehand (FH) and backhand lunges (BH) are two of the most critical forward lunge techniques (Hong et al., 2014; Hu et al., 2015; Valldecabres et al., 2018). In addition, due to the asymmetrical nature of badminton, players hold their racket with their dominant hand and maintain balance by adopting an asymmetrical posture. Different lateral limbs move in different movement patterns, so the effect of fatigued ankle dorsiflexors on the FH and BH may vary (Lin et al., 2015).

This study aimed to determine the effect of fatigued ankle dorsiflexors on the lower limb biomechanics of badminton players during FH and BH. This study hypothesized that fatigue of the dorsiflexor muscle groups would result in a decrease in ankle dorsiflexion angle, an increase in peak vertical ground reaction force (VGRF) and impact loading rates, and a decrease in dynamic postural stability in badminton players performing FH and BH.

## 2 Methods

### 2.1 Participants

The sample size was determined using data from previous studies. At least 15 participants were selected using G*Power3.1 with an alpha value of 0.05 and a power value of 0.80 and effect size of 0.80 (Hu et al., 2015; Nielsen et al., 2020; Lin et al., 2021). This study recruited 15 right-handed male professional badminton players with dominant right legs (Age: 23.30 ± 2.00 years; Body mass: 74.93 ± 3.98 kg; Height: 1.76 ± 0.02 m; Years of Experience: 5.90 ± 1.23 years) (Mei et al., 2017; Herbaut and Delannoy, 2020; Nielsen et al., 2020). Participants were selected on the basis of consistent badminton practice (at least 2 h per week) and at least 2 years of competition experience. Prior to the test, participants provided written consent and were informed of the testing procedures and requirements. Participants had no upper or lower extremity injuries in the previous 6 months. Subjects did not engage in high-intensity training or competition for 2 days before the experiment. The testers gave each participant the same type and brand of badminton shoes in order to eliminate the confounding effect of footwear (Fu, 2011; Mei et al., 2017). The local ethics committee approved the experiment (RAGH202108253005.7).

### 2.2 Experimental protocol and procedures

**FIGURE 1:**
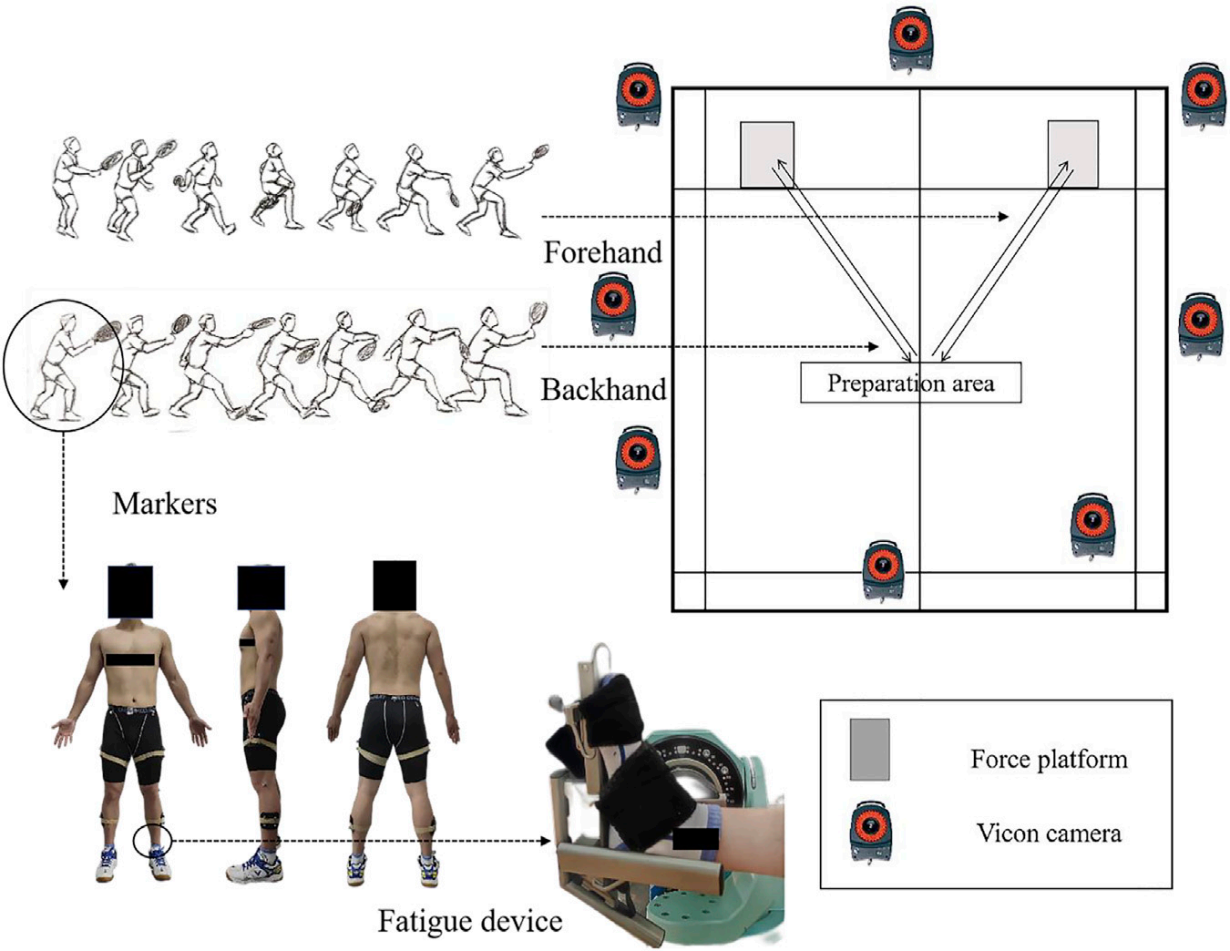
Illustration of experimental configuration and fatigue device.

Before the experiment began, the participants’ basic information (height and weight) was gathered. And the subject’s leg length (distance between the right anterior superior iliac spine and the outer ankle of the ankle joint) was measured to assist in the measurement of movement distance for each individual. Fatigue-inducing process: data collection prior to fatigue, the fatigue process, and data collection after fatigue. Before fatigue, static stance trials were conducted to determine the joint center and axis of rotation. Kinematic and kinetic data were gathered during the lunge Figure 1 shows the experimental design. According to previous studies, the forehand lunge is defined by moving in the direction of the racket hand, causing the chest to face the net, hitting the ball with the racket, and returning to the starting position as quickly as possible; each lunge should be completed within 3 s, with the lunge moving 1.5 times the length of the leg, whereas the backhand lunge has the back facing the net (Mei et al., 2017; Lam et al., 2018; Nielsen et al., 2020). Each participant completed a total of 10 successful lunges in both directions, five in each direction, with 30–60 s between each movement, with the FH and BH completed randomly.

After becoming familiar with the dorsiflexion fatigue task (dorsiflexion of the ankle at maximum ROM until fatigue), the participant laid supine on an isometric ergometer with fully extended knees. After sufficient movement of the ankle joint to warm up (to avoid muscle strain), participants performed three maximum isometric contractions in ankle dorsiflexion (120°/s) to determine the maximum peak moment that the participants can exert. After determining the maximum peak moment of the subject, a 4-min rest period was administered (Salavati et al., 2007; Boyas et al., 2011; Gautrey et al., 2013). The subjects were then instructed to repeat the dorsiflexion motion as rapidly as possible until they became fatigued. Fatigue of the dorsiflexor group is judged by three consecutive repetitions below 50 percent of the peak moment value (Yaggie and McGregor, 2002; Gribble and Hertel, 2004; Salavati et al., 2007; Boyas et al., 2011). During the fatigue process, participants were encouraged with positive cues to exert their maximum peak moment with each movement (McNair et al., 1996). After fatigued, kinematic and kinetic data on the lunge were collected.

### 2.3 Collection and processing

According to a previous study, 36 reflective markers (6DOF) were placed in the lower extremities and pelvis (Schafer et al., 2018; Xu et al., 2020; Jiang et al., 2021). The reflective markers are located at the big toe, the first and fifth metatarsal heads, the heel, the medial and lateral sides of the ankle, the middle of the tibia, the middle of the femur, the internal and external of the femoral condyles, the anterior superior iliac spine, and the posterior superior iliac spine. An eight-camera Vicon motion system (Oxford Metrics Ltd., Oxford, United Kingdom) was used to collect the kinematic data of the right lower limb of the subject during a lunge with a sampling frequency of 200 Hz. The C3D files generated by the Vicon Nexus software were imported into Visual 3D (c-motion Inc., Germantown, MD, United States) for further kinematic data processing. Embedded in the floor and synchronized with the Vicon system, an AMTI force plate (AMTI, Watertown, MA, United States) was used to collect kinetic data with a sampling frequency of 1,000 Hz. Kinematic and kinetic data were gathered during the lunge contact period, which was defined as the time between the impact of the dominant leg’s heel on the force plate and the withdrawal of the toe from the force plate (Lam et al., 2018). The kinematics and kinetics data were filtered with fourth-order zero-phase low pass Butterworth filters at 10 Hz and 20 Hz (Lam W.-K. et al., 2017; Jiang et al., 2021). An isometric muscle test device was applied to test the fatigue of the ankle dorsiflexor group (CON-TREX-MJ, PHYSIOMED, GER).

### 2.4 Statistical analyses

All analyses were conducted with SPSS 26.0 (SPSS Inc., Chicago, IL, United States) and MATLAB R2019a (The MathWorks, Natick, MA, United States). Max and Min for kinematic data are both the maximum and minimum values of the angles of the three joints in the sagittal and frontal planes during the lunge landing (by numerical comparison), and ROM is the difference between the maximum and minimum values (Max-Min). GRF data includes peak vertical ground reaction force, peak horizontal ground reaction force, maximum loading rate, and average loading rate (Kuntze et al., 2010; Lam W. K. et al., 2017; Lam et al., 2018). Briefly, the peak vertical ground reaction force and the peak horizontal ground reaction force were defined as the maximum value of the vertical and horizontal GRF, respectively (Lam et al., 2018). The maximum loading rate is the maximum slope of the VGRF curve between consecutive data points, ranging from 20% to 90% prior to the initial peak impact (Lam W.-K. et al., 2017; Lam et al., 2018), the mean loading rate is the average slope of the VGRF curve between consecutive data points, ranging from 0% to 100% prior to the impact of the initial peak (Lam et al., 2018). The displacement of the COP sagittal plane is the total offset of the COP on the *Y*-axis of the force table coordinate system, and the displacement of the frontal plane is the total offset of the COP on the *X*-axis of the force table coordinate system. Prior to statistical analysis, the Shapiro-Wilk test was used to examine the normality of discrete variables and sagittal and frontal waveform data of the ankle, knee, and hip joints. Pre-fatigue and post-fatigue data were compared using a paired-samples *t*-test; non-normally distribution data were examined using the Wilcoxon signed rank test, with a significance level of *p* < 0.05 and Bonferroni correction. The sagittal and frontal waveform data of the three joints not to be normally distributed (*p* < 0.05), hence Statistical Non-parametric Mapping (SNPM) was utilized to examine the waveform data of the ankle, knee, and hip joints.

## 3 Results

### 3.1 Lower limb joint angle and range of motion

**FIGURE 2:**
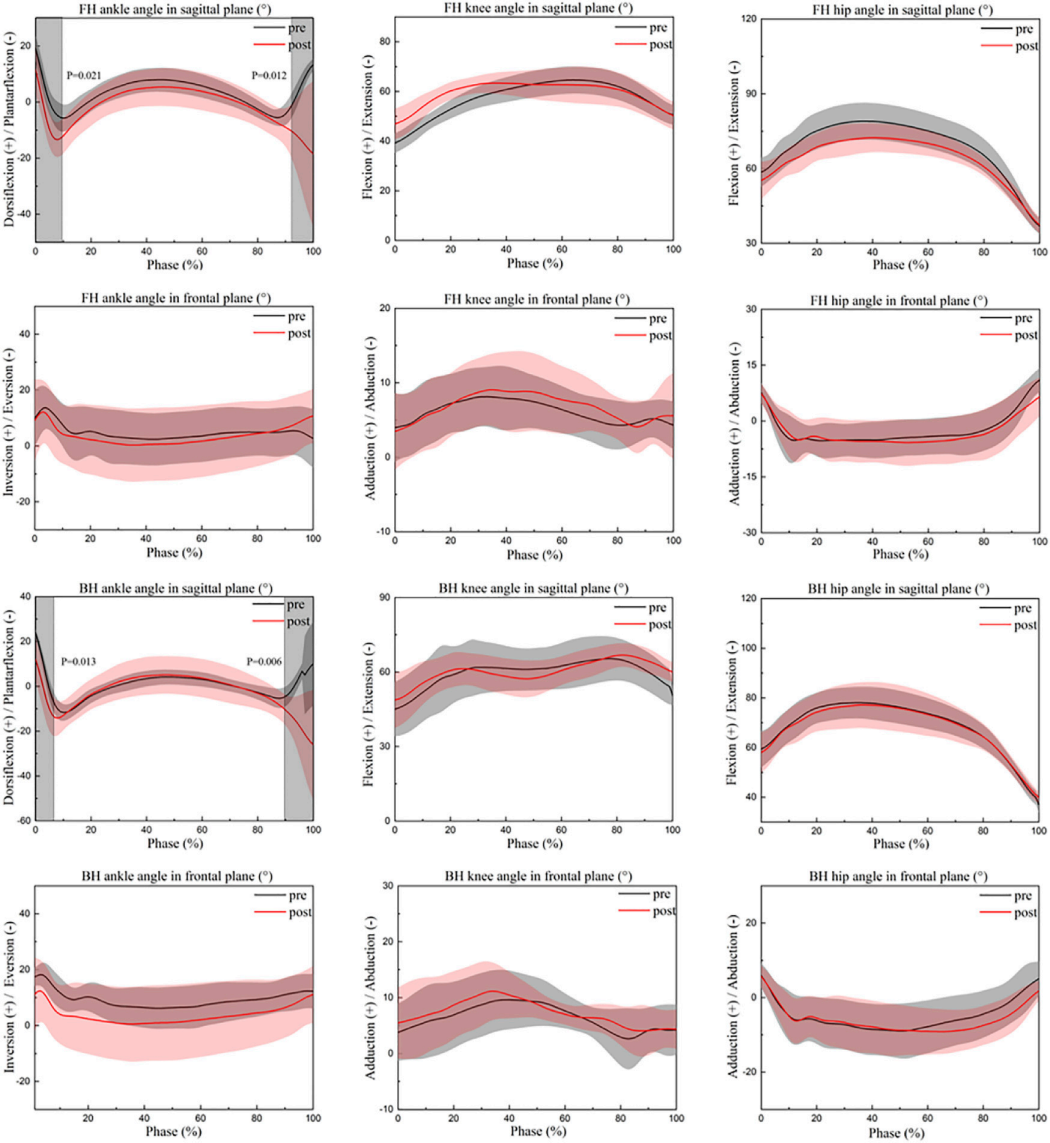
Changes in the lower limb angles in the sagittal-frontal plane during the strike phase. FH, forehand lunge; BH, backhand lunge; pre, pre-fatigue, post, post-fatigue.

**FIGURE 3:**
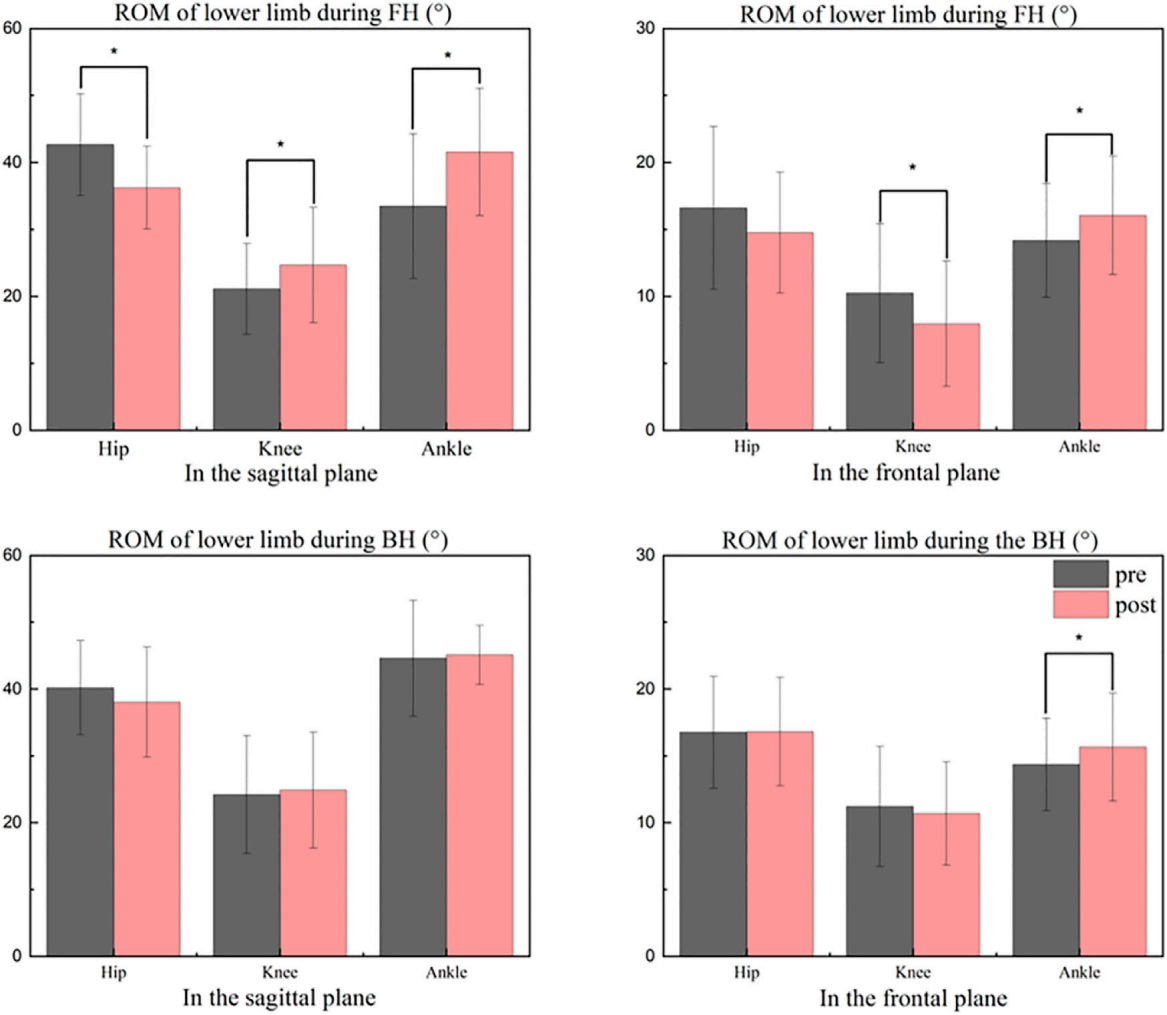
Comparisons between pre and after ROM levels during the strike period. FH, forehand lunge; BH, backhand lunge; pre, pre-fatigue, post, post-fatigue. *Significant differences at the hip, knee, and ankle (*p* < 0.05).

Table 1 shows the differences in joint angles and ROM in the sagittal and frontal planes between badminton players who did the FH and BH before and after their dorsiflexors got fatigued. Figures 2, 3 demonstrate the angle changes and ROM of the lower limb joints in the sagittal and frontal planes of badminton players who performed the FH and BH before and after their dorsiflexors became fatigued. Before and after fatigue of the dorsiflexor group, significant differences in ankle joint angles occurred between 0% and 10% (*p* = 0.021) and 93%–100% (*p* = 0.012) throughout the movement cycle when badminton players performed FH, and between 0% and 7% (*p* = 0.013) and 90%–100% (*p* = 0.006) during the BH (Figure 2).

**TABLE 1:** Comparison of pre-fatigue and post-fatigue means standard deviations for joint angles and ROM values during the strike phase of the FH and BH (unit: degrees).

Variables		FH					BH		
		Pre (°)	Post (°)	t/z	DF	Pre (°)	Post (°)	t/z	DF
Hip flexion (+)	Max	**79.59 ± 7.25**	**73.15 ± 5.78**	5.774	−6.44 ± 9.92	79.16 ± 6.73	77.84 ± 9.12	1.175	−1.33 ± 11.76
	Min	36.93 ± 3.20	36.89 ± 3.76	0.051	−0.04 ± 4.88	39.27 ± 2.61	39.69 ± 2.65	−0.920	0.42 ± 3.34
	ROM	**42.66 ± 7.64**	**36.26 ± 6.18**	5.210	−6.39 ± 9.41	40.18 ± 7.05	38.05 ± 8.23	1.967	−2.13 ± 11.68
Hip adduction (+)	Max	15.78 ± 8.39	13.88 ± 7.51	1.282	−1.9 ± 12.21	10.70 ± 7.73	9.55 ± 7.07	1.396	−1.15 ± 7.70
	Min	−0.81 ± 11.01	−0.90 ± 9.49	0.044	−0.09 ± 12.78	−6.30 ± 9.93	−6.52 ± 8.33	0.155	−0.22 ± 14.23
	ROM	16.60 ± 6.09	14.77 ± 4.52	1.551	−1.83 ± 8.32	16.77 ± 4.19	16.82 ± 4.06	−0.063	0.05 ± 5.87
Knee flexion (+)	Max	**55.73 ± 11.61**	**64.61 ± 6.90**	−5.600	8.88 ± 13.55	65.03 ± 12.64	69.87 ± 5.35	−2.482	4.84 ± 15.32
	Min	**34.93 ± 4.81**	**44.61 ± 5.07**	−10.042	9.68 ± 6.44	40.84 ± 8.70	44.97 ± 1.16	−2.143	4.13 ± 8.43
	ROM	**21.13 ± 6.79**	**24.70 ± 8.60**	−2.716	3.58 ± 9.89	24.19 ± 8.84	24.89 ± 8.65	−0.474	0.7 ± 11.41
Knee adduction (+)	Max	10.09 ± 5.68	10.82 ± 5.42	−0.600	0.72 ± 6.52	11.06 ± 6.02	12.05 ± 5.73	−1.062	0.99 ± 6.79
	Min	2.70 ± 3.92	0.21 ± 4.93	−1.839^a^	−2.49 ± 7.28	0.83 ± 5.66	1.00 ± 5.83	−0.710^a^	0.17 ± 6.81
	ROM	**7.96 ± 4.69**	**10.25 ± 5.18**	−2.336	2.28 ± 8.00	11.22 ± 4.51	10.69 ± 3.88	0.579	−0.54 ± 5.88
Ankle Dorsi-flexion (+)	Max	**15.06 ± 8.73**	**11.52 ± 9.56**	2.176	−3.53 ± 12.35	15.70 ± 9.04	12.75 ± 9.93	−1.250^a^	−2.95 ± 11.63
	Min	**−18.41 ± 16.89**	**−30.08 ± 15.26**	−3.403^a^	−15.02 ± 21.79	−28.90 ± 15.45	−32.35 ± 13.78	−0.989^a^	−3.45 ± 24.06
	ROM	**33.47 ± 10.82**	**41.59 ± 9.51**	−3.820	8.11 ± 13.51	44.60 ± 8.67	45.10 ± 7.25	−0.334	8.12 ± 15.29
Ankle Inversion (+)	Max	15.51 ± 8.32	15.61 ± 10.54	−1.028^a^	0.1 ± 13.12	**18.96 ± 8.26**	**15.44 ± 10.50**	3.840	−3.52 ± 9.93
	Min	1.47 ± 10.54	−0.70 ± 13.51	−1.300^a^	−2.17 ± 14.77	**4.64 ± 10.21**	**−0.75 ± 13.62**	4.542	−5.39 ± 11.02
	ROM	**14.18 ± 4.24**	**16.05 ± 4.42**	−2.191	1.86 ± 5.73	**14.36 ± 3.45**	**15.66 ± 4.04**	−2.517	1.3 ± 4.83

Note: FH, forehand lunge; BH, backhand lunge; Pre, pre-fatigue, Post, post-fatigue. Significant *p*-values (<0.05) are shown in bold. DF: The difference between post-fatigue and pre-fatigue.

^a^ Wilcoxon signed rank test results for non-normally distributed variables.

In the sagittal plane, peak ankle plantarflexion angle was significantly increased (*t* = 2.176, *p* = 0.034) and peak ankle dorsiflexion angle (*t* = −3.403, *p* = 0.001) were significantly reduced when athletes performed FH after fatigue of the dorsiflexor, hence ankle plantarflexion-dorsiflexion ROM was significantly increased (*t* = −3.820, *p* = 0.001) (Table 1). The peak knee flexion angle (Max: *t* = −5.600, *p* < 0.001; Min: *t* = −10.042, *p* < 0.001) and knee sagittal ROM (*t* = −2.716, *p* = 0.009) were significantly greater than pre-fatigue during the FH. Peak hip flexion angle (*t* = 5.774, *p* < 0.001) and hip sagittal ROM (*t* = 5.210, *p* < 0.001) were significantly reduced than pre-fatigue during the FH (Table 1). After fatigue, the peak knee flexion angle was significantly greater during BH than before fatigue (Max: *t* = −2.482, *p* = 0.017; Min: *t* = −2.143, *p* = 0.037) (Table 1).

In the frontal plane, after fatigue than that before fatigue of the dorsiflexors, the peak ankle inversion angle was significantly smaller than before fatigue when athletes performed BH (Max: *t* = 3.840, *p* < 0.001; Min: *t* = 4.542, *p* < 0.001), but the ankle frontal plane ROM was significantly greater than before fatigue (*t* = −2.517, *p* = 0.015) (Table 1).

### 3.2 Ground reaction force

**FIGURE 4:**
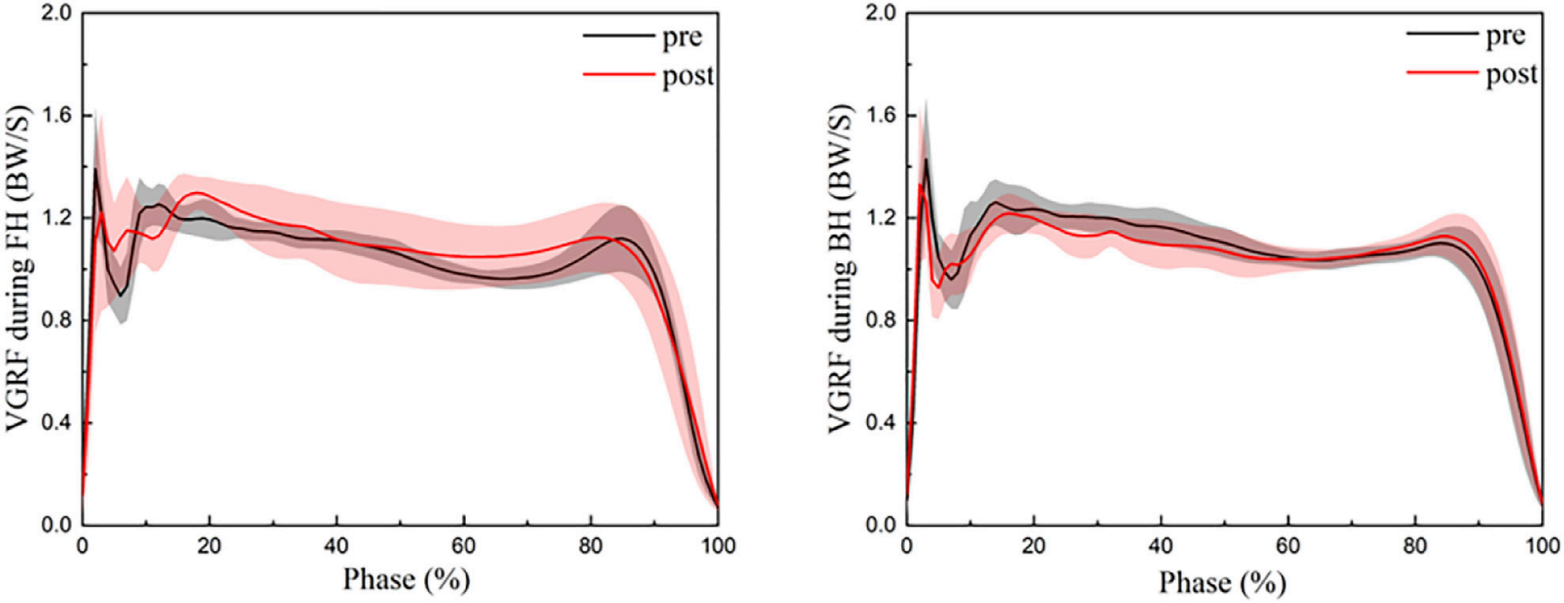
Change of the vertical GRF during the strike phase in the FH and BH. FH, forehand lunge; BH, backhand lunge; pre, pre-fatigue, post, post-fatigue.

Table 2 and Figure 4 illustrate the characteristics of GRF when an athlete performs FH and BH before and after dorsiflexor fatigue. Before fatigue of the dorsiflexor group, the peak horizontal reaction force of athletes performing FH was significantly lower than after fatigue (*t* = 3.721, *p* = 0.001) (Table 2). The maximum loading rate (*t* = −2.91, *p* = 0.005) and the mean loading rate (*t* = −3.531, *p* = 0.001) were significantly higher when the BH was performed by the athletes after dorsiflexor fatigue than before fatigue (Table 2).

**TABLE 2:** Comparison of pre-fatigue and post-fatigue means standard deviations for the ground reaction forces (GRFs) characteristics of the FH and BH.

Variables	FH					BH		
	Pre	Post	t	DF	Pre	Post	t	DF
Peak vertical GRF (BW)	1.55 ± 0.19	1.52 ± 0.22	0.717	−0.03 ± 0.31	1.50 ± 0.17	1.51 ± 0.22	−0.161	0.01 ± 0.30
Peak horizontal force (BW)	**−0.38 ± 0.06**	**−0.35 ± 0.06**	3.721	0.03 ± 0.10	−0.43 ± 0.09	−0.41 ± 0.09	−1.682	0.02 ± 0.11
Maximum loading rate (BW/S)	109.14 ± 31.70	103.58 ± 43.49	0.780	−5.56 ± 67.72	**103.10 ± 29.16**	**114.01 ± 31.55**	−2.911	10.90 ± 43.80
Mean loading rate (BW/S)	85.71 ± 27.50	80.79 ± 34.78	0.467	−4.92 ± 37.47	**78.74 ± 22.11**	**92.24 ± 27.23**	−1.682	13.5 ± 35.04

Note: FH, forehand lunge; BH, backhand lunge; Pre, pre-fatigue, Post, post-fatigue. Significant *p*-values (<0.05) are shown in bold. DF: The difference between post-fatigue and pre-fatigue.

### 3.3 Center of pressure

**FIGURE 5:**
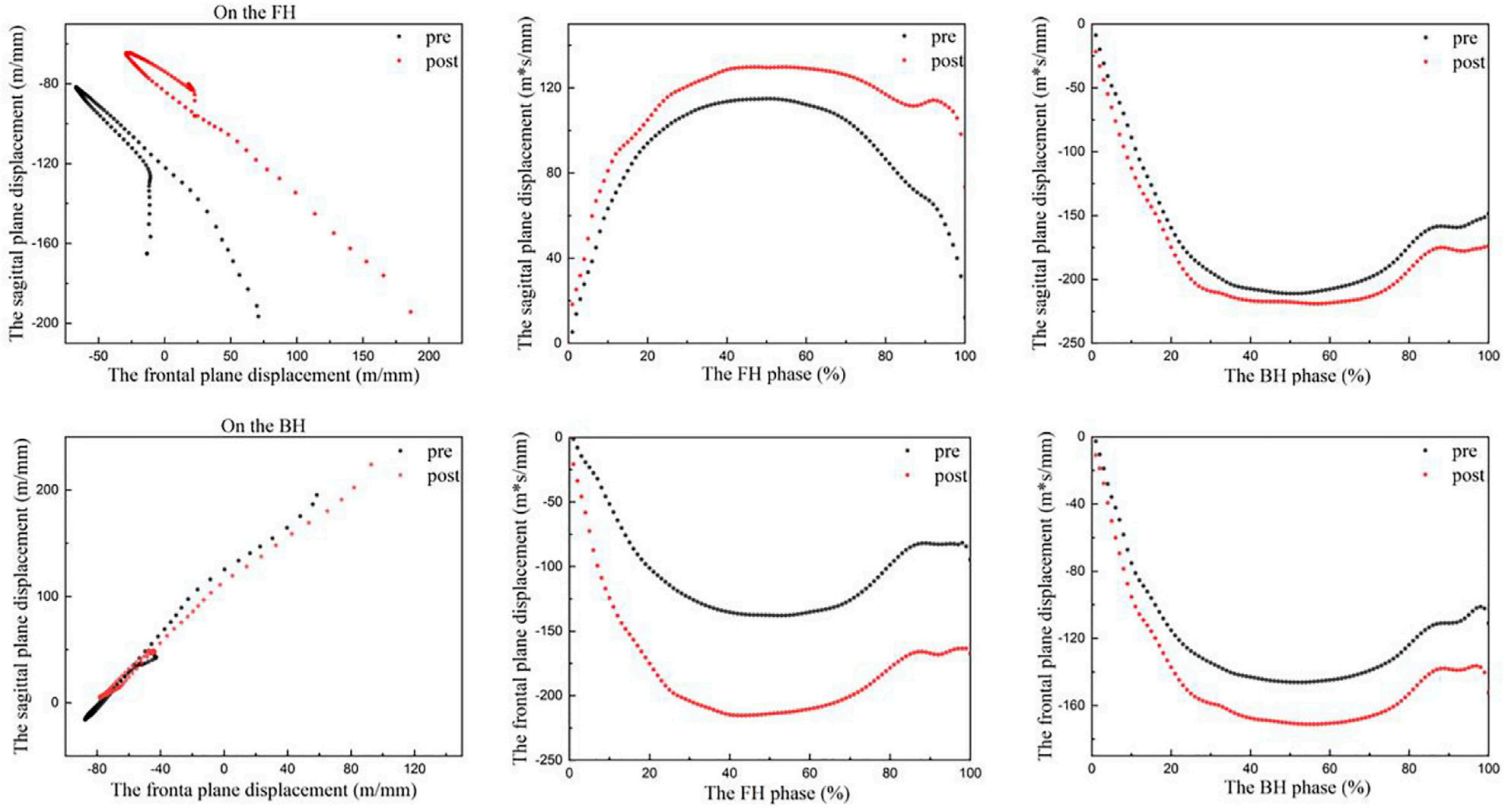
Change in the center of pressure in the FH and BH during the strike phase. FH, forehand lunge; BH, backhand lunge; pre, pre-fatigue, post, post-fatigue.

Table 3 and Figure 5 illustrate the pre-fatigue and post-fatigue COP displacement quantities in the sagittal and frontal planes during FH and BH. The frontal plane displacements during the FH and BH were significantly greater than before fatigue (FH: *t* = 4.394, *p* < 0.001; BH: *t* = 6.001, *p* < 0.001) (Table 3).

**TABLE 3:** Comparison of pre-fatigue and post-fatigue means standard deviations for center of pressure displacement characteristics in the FH and BH.

Variables	FH					BH		
	Pre	Post	t	DF	Pre	Post	t	DF
The Sagittal plane displacement (m*s/mm)	9.98 ± 3.19	10.85 ± 4.20	−1.381	0.88 ± 5.47	17.16 ± 2.34	17.64 ± 2.21	0.211	0.48 ± 2.87
The Front plane displacement (m*s/mm)	**16.21 ± 2.55**	**18.54 ± 2.70**	4.394	2.33 ± 3.69	**12.02 ± 2.36**	**14.40 ± 2.34**	6.001	2.39 ± 3.33

Note: FH, forehand; BH, backhand; Pre, pre-fatigue, Post, post-fatigue. Significant *p*-values (<0.05) are shown in bold. DF: The difference between post-fatigue and pre-fatigue.

## 4 Discussion

This study aimed to investigate the effect of ankle dorsiflexor group fatigue on the biomechanics of FH and BH in badminton players. Consistent with the hypothesis, after dorsiflexor fatigue, badminton players had decreased ankle dorsiflexion angle and decreased dynamic postural stability during FH and BH; impact loading rate increased significantly during BH. The VGRF and impact loading rate were not affected significantly before and after fatigue of the dorsiflexors during FH, did not support the hypothesis.

In badminton, it is critical to perform a great lunge and return to the starting position for the following stroke, which can affect the outcome of the entire game (Lam et al., 2020). Previous research has shown that fatigue negatively affects the quality of movement performance, whereas a substandard lunge landing increases the risk of lower extremity injury (Bulat et al., 2019; Buckthorpe, 2021). Thus, kinematic and kinetic data were gathered during the lunge landing for this study. According to previous research, a significant increase of 10° in ankle plantarflexion increases the tendency for calf muscle fatigue and overuse injuries in the foot (Lee and Loh, 2019), it may also induce Achilles tendon and anterior calcaneal ligament fatigue and damage (Fahlström et al., 1998; Fahlstrom et al., 2002; Fong et al., 2009; Mei et al., 2017; Lee and Loh, 2019). The previous study’s significant increase in plantarflexion may have been attributable to differences in skill level, with better badminton players employing a more efficient landing method (less plantarflexion). Consistent with previous studies, the current participants exhibited a significant increase in peak ankle plantarflexion angle of 11.5° after fatigue, which may be due to a significant decrease in peak ankle dorsiflexion angle of 3.5° and a significant increase in ankle sagittal ROM of 8° during FH. Participants exhibited less dorsiflexion, possibly due to dorsiflexor fatigue resulting in insufficient dorsiflexor muscle strength and relatively high plantarflexion strength. Under normal circumstances, moderate ankle valgus and foot pronation can help the lower extremity absorb vertical and rotational forces, which makes jumping and landing, running, and other activities less likely to cause injury (Dubin et al., 2011). Some studies have demonstrated a positive correlation between increased ROM in ankle valgus and a decreased risk of lower extremity injury (Hoch et al., 2015; Padua et al., 2019). In our study, the current participants showed a significant decrease in peak ankle valgus angle by 5° and an increased tendency to valgus after fatigue, which may be caused by an increase in peak plantarflexion angle by 3.4° and peak knee flexion angle by 4.8° during BH. Because when the ankle plantarflexion increases, the moment arm of the GRF increases and the ankle joint’s stability decreases; concurrently, due to the directional nature of the motion and the increased knee flexion, the trunk tends to move toward the left front, resulting in an increase in ankle valgus (Wright et al., 2000; Herbaut and Delannoy, 2020). An increase in ankle valgus ROM may provide sufficient cushioning to the lateral ankle collateral ligament. Therefore, this may be the body’s protective mechanism against harm. In addition, previous research revealed that amateur badminton players had a significantly greater ROM of 2.2 degrees of ankle inversion compared to professional badminton players (Fu et al., 2017). This was attributed to the lack of stability of the muscles surrounding the ankle joint in amateur players (Fong et al., 2009; Dubin et al., 2011). Such a change is apparently similar to our study, where participants showed a significant increase in ankle inversion ROM of 2.1° at FH and 1.3° at BH after dorsiflexor fatigue. This could be caused by the increased tendency of ankle valgus after fatigue. Hence, dorsiflexor fatigue may also affect the work of the unstable muscles around the ankle joint, which causes increased ROM in the frontal plane of the athlete.

Different degrees of knee flexion may result in varying movement performance and joint impact forces. It has been shown that elite badminton players show less knee flexion (9° less maximum flexion angle and 12° less minimum flexion angle) during the forehand lunge than recreational badminton players, enabling them to recover from their starting position and prepare for the next stroke more quickly (Mei et al., 2017). Interestingly, one study noted that during the lunge, athletes with knee injuries would reduce knee injuries and cushion the impact of landing on the joint by increasing knee flexion (2.8° compared to ROM in non-injured athletes) while boosting dynamic stability by lowering the center of mass (Huang et al., 2014). Again, in relation to our study, after dorsiflexor fatigue, the current subjects showed more flexed knee posture across the entire lunge in both FH and BH; a significant increase in knee flexion maximum of 8.9° and a minimum of 9.7° at FH; a significant increase in knee flexion maximum of 4.8° and a significant increase in minimum of 4.1° at BH. Such an increase is similar to previous studies. This may be because badminton players reduce the risk of injury by increasing knee flexion and decreasing athletic performance after dorsiflexor fatigue, which may be a neuromuscular protective mechanism of their own. Because it has been shown that at the moment of landing, each degree of knee flexion decreases the ground reaction force by 68 N, and greater knee flexion also increases the impact attenuation rate (Gerritsen et al., 1995; Lafortune et al., 1996; Duquette and Andrews, 2010). In the sagittal plane of the knee, greater flexion may be associated with a decreased risk of injury and a worsen in athletic performance, whereas the opposite may be true in the frontal plane. Studies have shown that the knee frontal ROM of elite badminton players is 4° greater than that of recreational badminton player. Moreover, studies have also shown that the frontal ROM of the knee joint of athletes with knee joint injuries is significantly larger than that of athletes without knee joint injuries by 3.4°. In addition, research has demonstrated that tiredness has no effect on the frontal ROM of the knee joint in badminton players. The reason for this phenomenon could be the difference in skill level or gender between the participants (Huang et al., 2014; Mei et al., 2017; Valldecabres et al., 2018). In general similarity to previous studies, the current subjects showed a significantly 2.3° increase in knee frontal ROM after fatigue, which may be the result of a cascade effect caused by a significant 4° increase in ankle frontal ROM during FH. According to previous studies, dorsiflexion of the ankle is negatively linked with knee frontal plane displacement (Sigward et al., 2008). When the range of dorsiflexion is decreased, the frontal plane compensatory motion of the ankle joint increases, which contributes to the movement of the tibia in the knee joint, resulting in an increase in the frontal plane motion of the knee joint (Gross, 1995; Baker and Juhn, 2000; Joseph et al., 2008). This will improve athletic performance and increase the dynamic stability of the knee joint, but it will also increase the loading on the knee joint (Huang et al., 2014).

In the lunge movement, the knee joint’s movement is closely linked to the hip joint’s movement. Previous research has shown that badminton players with knee injuries have less forward trunk movement (reduced trunk angle, that is the angle of the hip joint to the vertical axis) in order to reduce knee stress during the FH because of greater knee flexion (Huang et al., 2014). This phenomenon of reduced hip flexion (elite badminton players have a significantly greater peak hip flexion angle of 14° than recreational players) is also associated with good athletic performance, which can assist the player in returning quickly to the starting position (Handbook, 2014; Mei et al., 2017). Participants in our study had a significant reduction in hip ROM of 6.4° after fatigue, which might be related to a significant reduction in hip flexion angle at peak during FH of 6.4°. In combination with previous studies, the significant decrease in peak hip flexion angle can be explained by an increase in knee flexion and a shift of body weight forward because of fatigue (Lin et al., 2015; Leporace et al., 2020). During the lunge, the body employs compensatory strategies to alleviate the stress on the lower extremity joints by increasing knee flexion and decreasing hip flexion. Previous research has shown that the peak joint contact force and the ground reaction force develop proportionally to the distal-to-proximal extent of flexion of the lower limb joints, with the ankle joint, knee joint, and hip joint proportions being 2.63–2.75, 4.59–4.63, and 3.82–3.82, respectively (Zhang et al., 2000; Chen et al., 2020). As the major cause of energy dissipation, the knee extensors and hip extensors use eccentric contractions to relieve pressure on the lower limbs and preserve the dynamic balance of the movements as much as possible (Zhang et al., 2000).

In an excellent lunge, badminton players are subjected to ground reaction forces that are approximately 2–3 times their body weight, which greatly increases the stress on the lower limb joints (Lam et al., 2018). The magnitude of the impact loading rate is positively correlated with the risk of stress fracture (Milner et al., 2006; Hansen et al., 2008; Puddle and Maulder, 2013). In a previous study, the vertical ground reaction force and loading rate were not significantly different before and after fatigue (Valldecabres et al., 2018). In contrast, the current participants had a significant increase in maximum loading rate of 11 BW/S and an average loading rate of 13.5 BW/S during FH after fatigue in our study. This may be a result of the differences generated by various fatigue schemes. Previous research on fatigue schemes focused on overall fatigue, but ours focused on local fatigue. The significant increase in loading rate may be the result of a decreased ankle dorsiflexion range, which reduces the body’s ability to absorb high impact forces during fatigue and increases the risk of stress fractures in the lower limbs (Lee et al., 2018; Radcliffe et al., 2021). Common knee injuries in badminton, such as anterior cruciate ligament (ACL) injuries, can be the result of repeated high impact stresses (Shariff et al., 2009; Lam et al., 2018). Horizontal reaction force is closely related to it, and in a lunge to resist a greater horizontal reaction force, eccentric contraction of the knee extensors increases and knee flexion increases, resulting in ACL overuse (Chappell et al., 2005; Lam et al., 2018). After fatigue, the current participants showed a significant decrease in peak horizontal reaction force of 0.03 BW, which corresponded to an increase in knee flexion. However, this is not conducive to kicking off the ground and returning to the starting position late in the lunge (Fukashiro et al., 1995; Kuntze et al., 2010).

The existence of better postural control is also seen as an indirect predictor of better athletic performance and a lower injury propensity (Edis et al., 2016; Pau et al., 2019). Additionally, the flexibility of the ankle joint and the muscle strength of the ankle and knee joints influence dynamic postural stability (Williams et al., 2016). After fatigue, the current participants showed a significant increase in frontal plane displacement of 2.3 m*s/mm during FH and 2,4 m*s/mm during BH. This may be caused by fatigue of the dorsiflexors leading to increased ROM of the frontal plane of the ankle and a tendency to ankle valgus, which results in a significant increase in body displacement in the left-right direction and less body control of posture than before fatigue (Padua et al., 2019). Thus, dorsiflexor fatigue may also negatively influence dynamic postural stability.

The study presented in this paper also has some limitations. At first, the participants were professional badminton players. In future studies, it should be considered whether experimental results (e.g., different athletic performance, ground reaction forces, etc.) occur before and after fatigue in amateur players that differ from those of professional players. Second, this study only looked at men, and gender differences should be taken into account in future studies. Third, mental and metabolic fatigue can also have an effect on muscle fatigue. What does this effect look like? How big is the effect? It is unknown as well. In addition, there are constraints in the recovery of local muscle fatigue (speed of recovery, etc.) that I hope future researchers will take into consideration.

## 5 Conclusion

The results of the study showed that when badminton players performed forehand lunges, more significant changes in lower limb joint angles and ROM occurred before and after fatigue of the dorsiflexor group. Fatigue may have a profound effect on the FH performance of badminton players, this may be a compensating mechanism employed by the body to reduce the risk of damage. After dorsiflexor fatigue, badminton players performed the backhand lunge with a subtle change in joint angle and range of motion, while the impact loading rate increased significantly. When developing a training program, it should be considered to enhance the training of the dorsiflexors of the ankle joint. Badminton players in a state of dorsiflexor fatigue can minimize the use of the backhand lunge in order to reduce the occurrence of injury. But forehand lunge sports performance is more negatively affected by fatigue. The advantages and disadvantages of performing these two movements after fatigue are pointed out for the athletes.

## Data Availability

The raw data supporting the conclusion of this article will be made available by the authors, without undue reservation.
